# Cumulative effects of heat exposure and storage conditions of Oxytocin-in-Uniject in rural Ghana: implications for scale up

**DOI:** 10.9745/GHSP-D-14-00043

**Published:** 2014-07-10

**Authors:** Luke C Mullany, Sam Newton, Samuel Afari-Asiedu, Edward Adiibokah, Charlotte T Agyemang, Patience Cofie, Steve Brooke, Seth Owusu-Agyei, Cynthia K Stanton

**Affiliations:** a Johns Hopkins Bloomberg School of Public Health, Baltimore, MD, USA; b Ghana Health Service, Kintampo Health Research Centre, Kintampo, Ghana; c Program for Appropriate Technology in Health (PATH), Seattle, WA, USA

## Abstract

**Objective:**

Postpartum hemorrhage can be reduced substantially in home deliveries attended by community-based workers by using Oxytocin-in-Uniject (OIU) devices affixed with temperature-time indicators. We characterized the distribution of time to discard of these devices when stored under normal field conditions in Ghana.

**Methods:**

Two drug storage simulation studies were conducted in rural Ghana in 2011 and 2012. Devices were transported under refrigeration from manufacture (Argentina) to storage at the study site. Twenty-three field workers each stored at home (unrefrigerated) 25 OIU devices and monitored them daily to record: (1) time to transition from usable to unusable, and (2) continuous digital ambient temperature to determine heat exposure over the simulation period. Time to discard was estimated and compared with mean kinetic temperature exposure of the devices during the shipment and storage phases and with characteristics of the storage locations using Weibull regression models. We used the time to discard distributions in a Monte Carlo simulation to estimate wastage rates in a hypothetical program setting.

**Results:**

Time for shipment and transfer to long-term refrigerated storage and mean kinetic temperature during the shipment phase was 8.6 days/10.3°C and 13.4 days/12.1°C, for the first and second simulation studies, respectively. Median (range) time to discard when stored under field conditions (unrefrigerated) was 43 (6 to 59) days and 33 (14 to 50) days, respectively. Mean time to discard was 10.0 days shorter in the second simulation, during which mean kinetic temperature exposure was 3.9°C higher. Simulating a monthly distribution system and assuming typical usage, predicted wastage of product was less than 10%.

**Conclusion:**

The time to discard of devices was highly sensitive to small changes in temperature exposure. Under field conditions typical in rural Ghana, OIU packages will have a half-life of approximately 30 to 40 days based on the temperature monitor used during the study. Program managers will need to carefully consider variations in both ambient temperature and rate of use to allocate the appropriate supply level that will maximize coverage and minimize stock loss.

## Abstract (precis)

Oxytocin-in-Uniject devices could be stored 30 to 40 days without refrigeration under typical field conditions, with wastage levels below 10%, based on simulation studies.

## INTRODUCTION

Oxytocin has been included on the World Health Organization (WHO) *Model List of Essential Medicines* since it was first published in 1977,^1^ and it is the drug of choice to prevent and treat postpartum hemorrhage—the leading cause of maternal death globally.^2^ There is considerable international commitment to expanding access to this drug, as reflected in recent recommendations from the UN Commission on Life-Saving Commodities for Women's and Children's Health. These recommendations include developing effective global mechanisms for pooled procurement and aggregated demand; supporting at least 3 manufacturers to develop and market high-quality, competitively priced oxytocin; and standardizing national regulatory processes for oxytocin registration.^3^

An important constraint to expanding access is that oxytocin is not heat stable, a key consideration in peripheral settings in low-income countries where consistent and reliable cold-storage capability is often lacking.^4^ This factor discourages health planners from procuring oxytocin for peripheral sites and could lead to waste and/or discarding of potentially usable oxytocin due to concerns about loss of potency. On the other hand, lack of heat stability can lead to sale and use of poor-quality oxytocin; for example, 74% of oxytocin samples purchased at private pharmacies in Ghana were outside manufacturer specification for active pharmaceutical ingredient (API).^5^

Oxytocin is the drug of choice to prevent and treat postpartum hemorrhage, but access has been constrained because it needs to be refrigerated.

Current *International Pharmacopoeia* guidelines recommend storing oxytocin at 2°C to 8°C and protecting it from light exposure, but the drug can withstand moderate heat exposure.^6^ A simulated temperature exposure study demonstrated that when exposed to either low (4°C to 8°C) or high (30°C) temperature continuously for 12 months, oxytocin retained 100% and 86% of its API, respectively, and was not affected by light exposure.^4^ The authors recommended that all oxytocics be refrigerated to the extent possible but that temporary storage up to 30°C for 3 months was acceptable.

**Figure 1.**
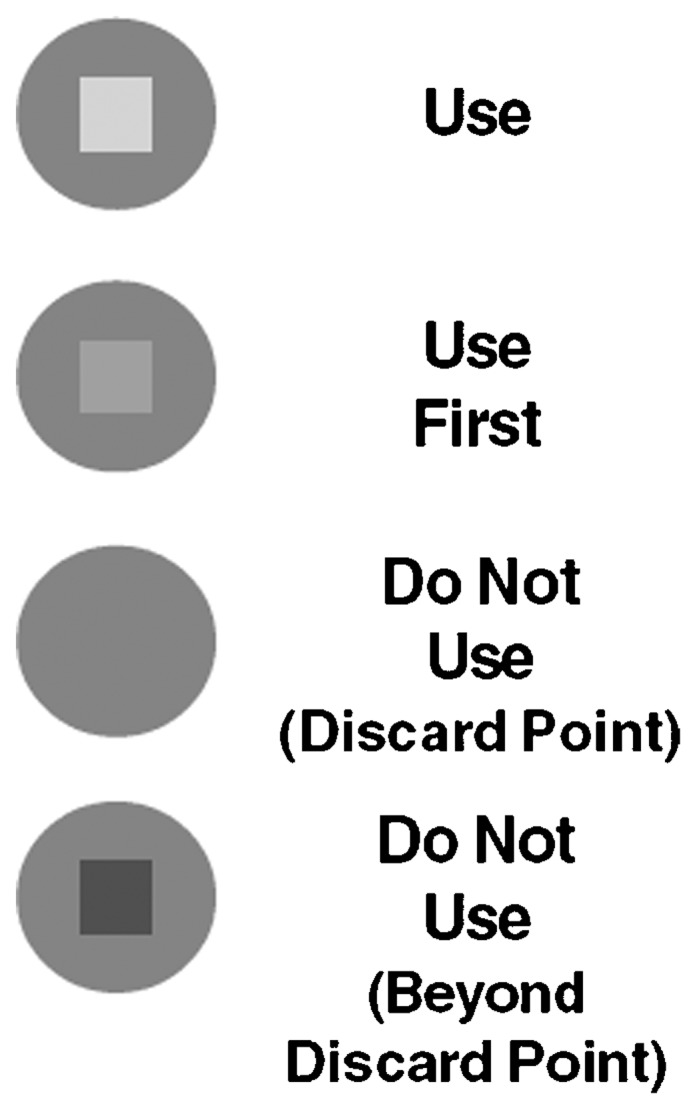
Visual Changes to the Temperature-Time Indicator Resulting From Cumulative Heat Exposure

One possible strategy for extending access to oxytocin to the peripheral health system would be to store the drug under cold-chain conditions at regional drug depots and health facilities with reliable refrigeration (as used for vaccines), and then for community-based providers to maintain a smaller temporary supply outside the cold chain, only accessing the refrigerated stock periodically for resupply. For this approach to be successful, the community-based providers would need to know the point at which an individual ampoule or injection device for oxytocin should be discarded. This would depend on cumulative exposure to heat, which would be expected to vary under different field conditions. Temperature-time indicators (TTIs) that change color in response to cumulative heat exposure provide a solution for this issue. The TTI is a simple label depicting a square enclosed in a circle (Figure 1); as the TTI (and therefore the drug) is cumulatively exposed to heat, the inner square begins to darken (a signal to prioritize use of this drug first), and then reaches the same color (or darker) than the outer circle, signaling that the drug should be discarded.

Temperature-time indicators change color in response to cumulative heat exposure, providing a way for health workers to monitor usability of oxytocin kept outside the cold chain.

We recently completed a cluster-randomized trial of the effect of prophylactic use of Oxytocin-in-Uniject (OIU) on postpartum hemorrhage among home deliveries, which found that OIU reduced postpartum hemorrhage by half in home deliveries in Ghana.^7^^,^^8^ OIU is a prefilled, sterile, non-reusable injection device with a single, 10 IU dose of oxytocin. In that trial, we conducted a sub-activity to systematically assess the duration that oxytocin can be stored outside the cold chain based on use of a TTI that had been applied to the foil envelopes containing the OIU devices. Specifically, we aimed to estimate the overall distribution and median time to reaching the discard point when oxytocin is removed from the cold chain and stored under realistic field conditions in rural Ghana.

The specific TTI used in this study, and that has been used previously for oxytocin, was designed to reach the discard point at the equivalent of 17.2 days at 40°C, 72.5 days at 30°C, or 154 days at 25°C. The choice of this particular TTI is conservative, as oxytocin exposed to these heat equivalents could actually remain within API specifications for longer time periods. Because the TTI was designed for monitoring vaccine vials, it does not function correctly when exposed to ultraviolet light and thus, if used to monitor oxytocin, requires storage in darkness.

## METHODS

This study was a sub-activity of a larger cluster-randomized trial of the impact of OIU on postpartum hemorrhage among women delivering at home in the Brong-Ahafo region of Ghana, the design^7^ and results^8^ of which have been published previously. In the randomized trial, OIU devices (with TTI) were distributed to community health officers (CHOs) on a regular basis guaranteeing that they would have a sufficient supply when called (by mothers/family members) at the time of labor and delivery. The CHOs maintained their supply of OIU without refrigeration in their assigned Community-based Health Planning and Services (CHPS) compound. During trial implementation, the CHOs used only those devices for which the TTI indicated that the drug had not yet reached the discard point.

### Overview of the Simulation Study

We were interested in systematically characterizing the distribution of time to which the TTI reached the discard point. During a pre-simulation phase between 2010 and 2011, we monitored the state of the OIU devices and the temperature conditions during storage from the time between manufacture and shipping to our storage facility at the Kintampo Health Research Centre (KHRC) in Kintampo, Ghana, and during storage at that site. We then conducted a simulated storage activity between 2011 and 2012 by asking field workers to store a quantity of devices in their homes while monitoring once daily the status of the TTI. Ambient temperature was monitored using a LogTag device, a battery-operated, digital ambient temperature recorder. The simulation exercise was conducted twice using different shipments of OIU devices during 2 different calendar periods, representing seasonal variation in rural Ghana. We also collected some basic information regarding the characteristics of field workers' homes and how they stored the devices out of refrigeration.

### Pre-Simulation: Temperature Monitoring From Manufacture to Arrival at KHRC

Prior to shipment from Biol, the Argentina-based manufacturer of the OIU used in this study, LogTag devices were placed in the containers used to transport the OIU in order to monitor the temperature to which the OIU devices were exposed during shipment from manufacturing site to storage at the KHRC. Two such pre-simulation studies were conducted on separate batches of OIU devices. Devices used in the first pre-simulation were shipped in August 2010, while the second set of devices was shipped in November 2011. In each shipment, there were 2 separate containers; within each was packed a separate LogTag, and these devices were synchronized to record at the same time points and with equal recording interval (every 30 and 15 minutes for the 2010 and 2011 shipments, respectively). For both batches, OIU devices were manufactured and stored at Biol, air transported under refrigeration from Buenos Aires, Argentina, to Johannesburg, South Africa, to Accra, Ghana. Upon arrival in Ghana, the containers remained at the airport (a non-climate-controlled environment) through customs processing, before being transported by road to the KHRC in Kintampo (a distance of about 450 km), and being returned to refrigeration upon arrival at the center. The LogTag collected data continuously from preparation of the shipment from Biol until placement of the OIU devices in controlled refrigeration at KHRC, where they were stored at 2°C to 8°C until received by field workers for the simulation exercise.

### Simulated Field-Based Storage Exercise

Starting in July 2011, and again in February 2012, we exposed packages of OIU devices to continuous ambient temperatures in the community. To avoid wastage, the OIU device itself was removed from its package, because we were interested only in monitoring the time for the TTI that was adhered to the package to reach the point of discard.

We chose 23 field workers to conduct this simulation and collect the monitoring measurements. All field workers were KHRC workers actively engaged in the parent randomized trial within which this activity was nested. We provided each field worker with 25 packages stapled to a piece of cardboard for easy viewing. In order to prevent direct exposure to light, the board was folded and placed in a small backpack during storage in the field worker's house. Each OIU package was pre-labeled with a unique identifying number.

Workers used a calendar data collection form into which he/she recorded the status of the TTI on a daily basis with 1 of 3 values:

- “1” (inner square lighter than outer circle)
- “2” (inner square same color as outer circle)
- “3” (inner square darker than outer circle)

A value of “2” corresponds to the discard point of interest in this study. In addition to the 25 devices, also included in each backpack was a LogTag device, set to record the temperature within the storage container at regular intervals. The temperature was recorded in degrees Celsius every 10 minutes and every 23 minutes in the first and second rounds, respectively. Field workers checked the status of their packages each morning and continued doing so until the TTI for all 25 packages on their board had reached the discard point.

### Characterization of Storage Locations

Basic information about each field worker's storage location was collected. These data included the district (Kintampo North vs. South); GPS waypoints; the storage location within the household; exposure of the backpack (in which the OIU packages were stored) to direct sunlight; construction materials used for roof, ceiling, wall, and floor; presence of fan; and whether the field worker cooked in the same room in which the devices were stored.

### Sample Size

We calculated the number of devices required to estimate with 95% confidence the median time to discard within 1 week (+/− 7 days). To do so, we assumed that the probability of discard increases with time, and thus anticipated modeling the time to discard using a Weibull distribution; without prior knowledge we assumed the mean time would be 180 days with a shape parameter of 3. For sample sizes ranging from 100 to 1,000, we simulated 10,000 datasets distributed as Weibull with these parameters, and we estimated the proportion of datasets resulting in median discard times within 7 days of the median parameter; this proportion reached 95% when the sample size was 580. Therefore, we distributed 25 OIU packages to each of 23 field workers for a total of 575 OIU packages.

### Analysis

The pre-simulation data (data on storage and handling of the OIU devices from manufacture to shipping to the storage facility at KHRC) were analyzed as follows: For each time point during transport, the exposure temperature was defined as the average temperature recorded by the 2 LogTag devices at that time point. The curve of temperature exposure for the first and second shipments was plotted over the time of shipment. In order to visualize and compare across the 2 shipments, the x-axis (in units of days) was standardized such that elapsed time was relative to the moment of shipment from Biol. Mean kinetic temperature for each shipment was calculated over the period of shipment using the batch-specific interval temperature measurements.

For the simulation exercise, the date at which the field worker received the test packages was defined as time 0 (T_0_). For each package, the time of discard, T_f_, was defined as the date that the field worker first recorded a switch from “1” to “2” (that is, when the inner square reached the same color as the outer circle), and the difference between T_0_ and T_f_ indicated the time to discard. The Kaplan-Meier estimate of the survival (discard) function was constructed, and the median and 95% confidence interval (CI) were estimated. Individual scatter plots of ambient temperature exposure across the time period of the simulation exercise were constructed. For each package, a measure of ambient temperature exposure was estimated by calculating the mean kinetic temperature from individual daily estimates of minimum/maximum temperature. To determine the effect of ambient temperature exposure and to examine other variables such as shipment (first vs. second), district (Kintampo North vs. South), and characteristics of the storage location, we estimated hazards ratios using a Weibull survival regression model and accounted for possible correlation within storage location using the Huber-White sandwich estimator. Mann-Whitney tests were used to compare the distribution of summary measures (mean, standard deviation, maximum, minimum) of temperatures to which each of the 23 groups of 25 devices were exposed during Phase 1 and Phase 2 with selected characteristics of the storage location, including roof and wall materials, presence of ceiling or a fan in the room, and whether the storage room was also used for cooking. Those found significant were then examined for their association with time to discard in Weibull regression models. We conducted a Monte Carlo simulation study using the estimated distribution of time to discard, in order to estimate the wastage that might occur in a hypothetical scaled-up program with monthly distribution and provision of a 10% buffer (excess supply beyond predicted actual use). All analyses were done with STATA 12.1.

## RESULTS

### Pre-Simulation Studies

For each of the 2 batches included in the pre-simulation study, the transportation period consisted of 6 distinct stages:

1. En route from manufacturer to Buenos Aires
2. En route to South Africa
3. En route to Ghana
4. Customs and storage in Accra, Ghana
5. En route to KHRC
6. Storage in KHRC

**Figure 2.**
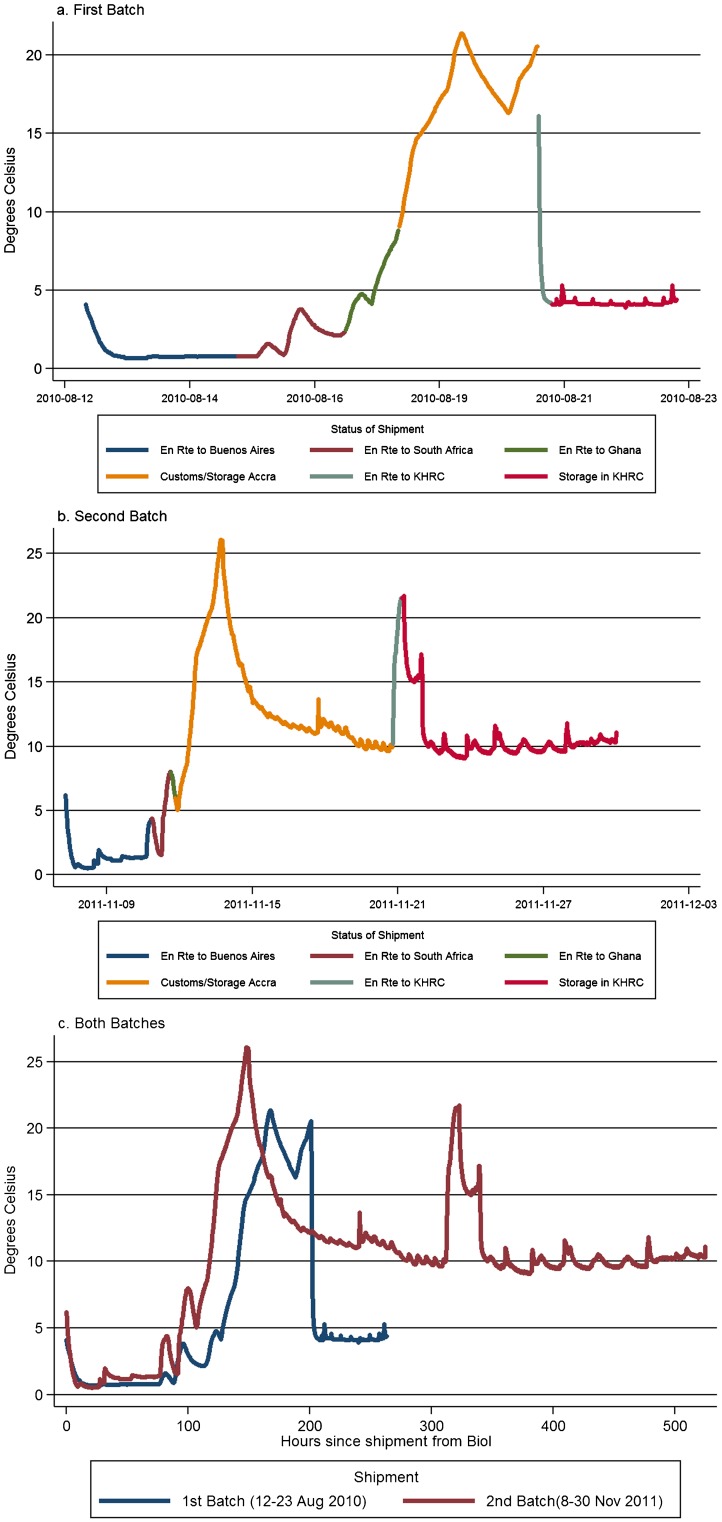
Average Ambient Temperature Exposure During Shipment Period, by Batch Abbreviation: KHRC, Kintampo Health Research Centre.

The pattern of fluctuation in temperature to which the devices were exposed during these 6 transportation stages was similar between batch 1 (Figure 2a) and batch 2 (Figure 2b), but overall exposure was higher in the second batch (Figure 2c), mainly due to higher temperature during the road transport stage. Mean kinetic temperature exposure prior to reaching long-term storage in the KHRC refrigerators was 10.3°C and 12.1°C in batch 1 and batch 2, respectively. Total duration from shipment to storage in KHRC for the first and second pre-simulation studies was 8.6 and 13.4 days, respectively.

Mean kinetic temperature/time exposure between shipment of the devices and storage at the central facility was 10°C–12°C /9–13 days.

### Simulation Study

The first simulation study started on July 25, 2011. Among 23 field workers initially provided with 25 OIU packages each, TTI status data were available for 550 of the 575 packages; information for all 25 packages for 1 field worker was missing for 1 month, during which all the indicators reached the discard point, thus the discard time could not be determined.

**Figure 3.**
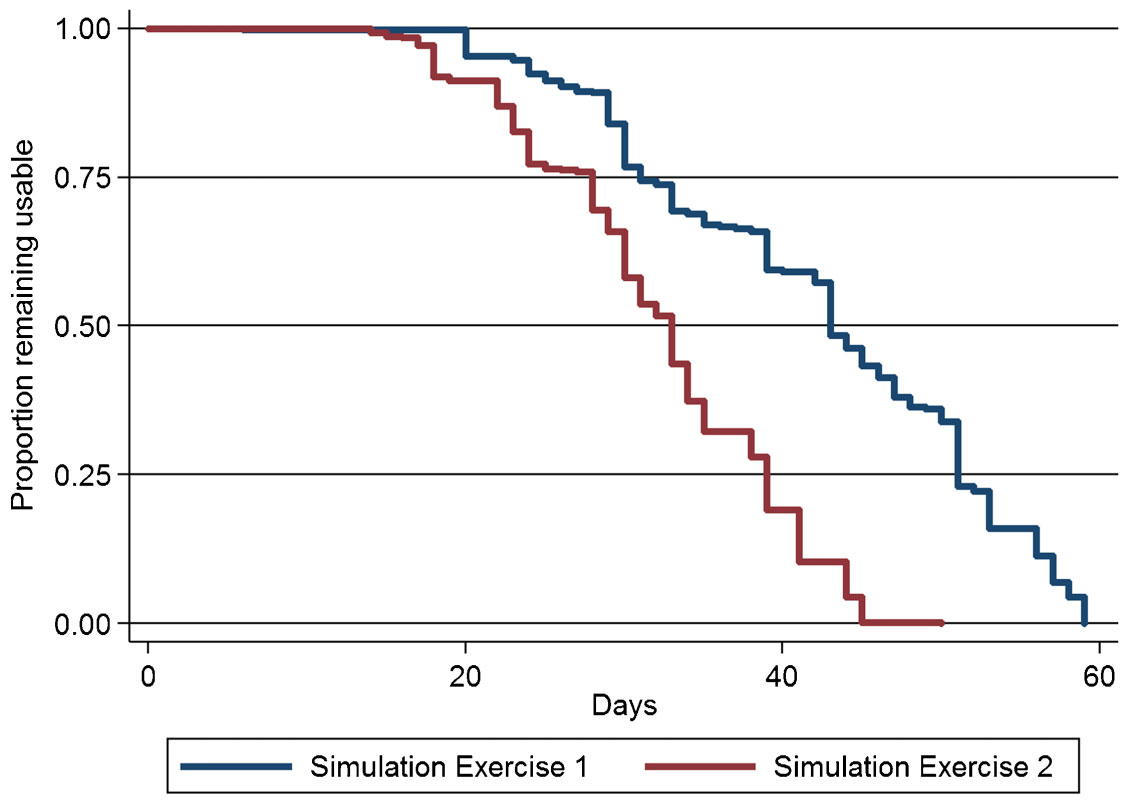
Proportion of Packages With TTI Status of Usable Over Time, by Simulation Exercise Abbreviation: TTI, temperature-time indicator.

Among the 550 packages with analyzable data, the durations at which the TTI indicators reached the discard point ranged from 6 to 59 days, and the median time was 43 days (95% CI = 43–45) (Figure 3). At 30 days, a common interval for community-based resupply of materials, 16% (88/550) of the packages had reached the discard point. Mean kinetic temperatures recorded across the 23 field workers' storage sites during the first simulation ranged from 25.1°C to 28.5°C.

The second simulation study used OIU packages from the second shipment batch and was started on February 1, 2012. Among 23 field workers provided with 25 OIU packages each, information from this simulation exercise was available for all 575 packages. The durations at which the TTI reached the discard state during the second simulation study ranged from 14 to 50 days, and the median time was 33 days (95% CI = 31–33). At 30 days, 34% (196/575) of packages reached the discard state. Mean kinetic temperatures recorded across the 23 field workers' storage sites during the second simulation ranged from 27.6°C to 33.0°C.

Median time to discard was 43 days and 33 days, in the first and second simulation studies, respectively.

**Figure 4.**
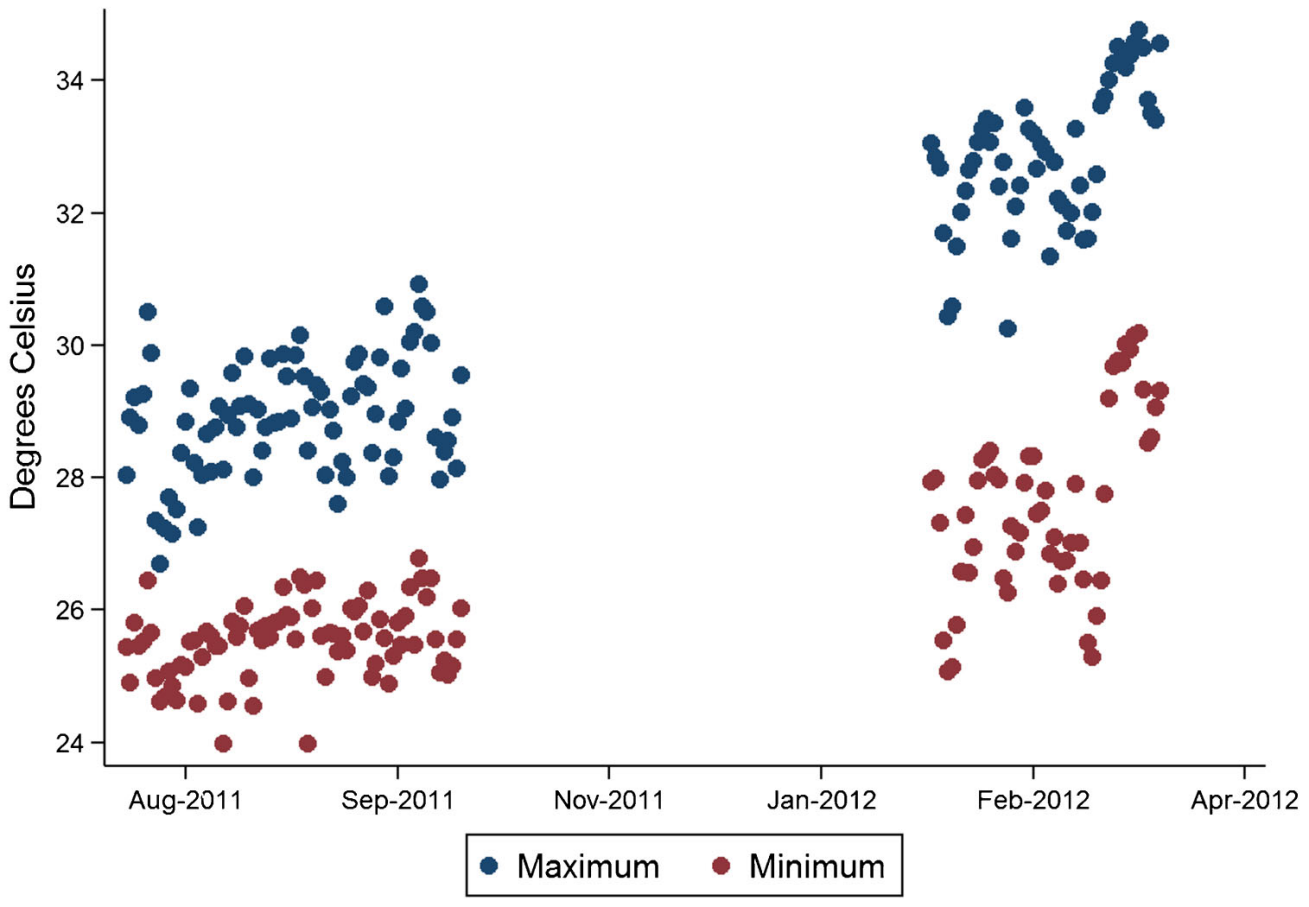
Daily Maximum and Minimum Temperature, by Day, for First and Second Simulation Exercises

The mean time to reaching discard was 10.0 days (95% CI = 4.4–15.7) shorter in the second study than in the first, and the hazard ratio of reaching discard was approximately 3.64 times higher (95% CI = 2.03–6.52). This was, however, nearly fully explained by the difference in ambient temperature exposure between the 2 simulation periods (Figure 4). The average maximum daily temperature across all OIU packages was approximately 3.9°C (95% CI = 3.6–4.3) higher during the second study period (32.8°C) compared with the first study period (28.9°C). Each degree increase in mean kinetic temperature increased the hazard of discard by 1.60 (95% CI = 1.28–2.00).

**Figure 5.**
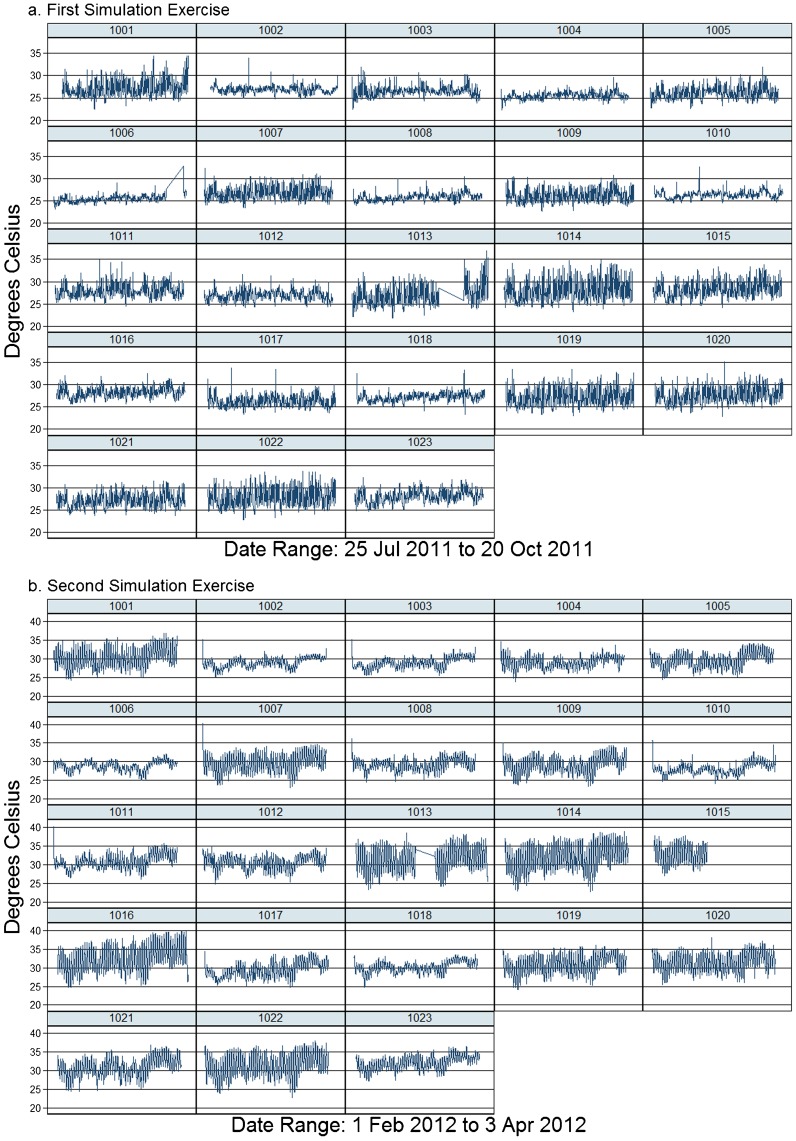
Scatter Plots of Temperature Readings by LogTag Device

Individual OIU packages were exposed to a wide range of temperatures, and, in some cases, there was substantial variation in exposure temperatures by field worker, illustrated with scatter plots of the individual LogTag measurements collected by each of the 23 workers in the first (Figure 5a) and second studies (Figure 5b). Differences in temperatures were also seen by district, with the northern part of the study area having consistently higher average exposure temperature than the southern portion.

All 23 workers reported that they were able to store their packages out of direct sunlight within their homes. None of the household characteristics examined were associated with mean, maximum, or minimum temperatures measured. However, *variation* in temperature was significantly higher among the 13 workers reporting that they had no ceiling (compared with 10 others reporting either a plywood or mat ceiling). Concurrently, many of these same 13 workers indicated that they felt their room became very hot because they did not have a ceiling. The hazard of discard was approximately 1.60 times higher (95% CI = 0.98–2.64) for devices stored in homes without a ceiling than in those with a ceiling.

In our Monte Carlo estimation, frequency of supply was set at 30 days (monthly distribution), and quantity of supply was sufficient to cover expected usage plus a 10% buffer. Using the time to discard distributions of the first and second field simulation exercises, the expected wastage rate was 3.5% and 8.8%, respectively.

## DISCUSSION

The results of these 2 simulation activities indicate that in rural Ghana, the median time between distribution to field workers and transition to a non-usable state for Oxytocin-in-Uniject (based on the TTI in current use) is on the order of 30 to 40 days (4 to 6 weeks) and is highly dependent on ambient temperature. Using a commonly used monthly resupply schedule, the number of devices discarded would be very low if the true discard time followed a distribution similar to that observed in this study, because a substantial proportion of devices would be used (rather than simply stored) before reaching discard. It is important to note that our simulation study did not include cold-chain storage at the final stage (that is, with the field workers), as is typical of many low-resource settings. However, we acknowledge that the cold chain was maintained at our central site, prior to distribution to the field workers, which in some instances may not be achieved in program settings.

The number that would be lost to heat exposure is a complex interaction between the rate of use, the excess supply provided (the proportion provided to the worker that is beyond the expected number that will be used), and the specific distributional characteristics of the time to discard. Our data from the first and second simulation exercises indicate that 16% and 34% of packages, respectively, reached the discard point within 30 days when not being used (that is, they were stored only). It is essential to note, however, that the actual wastage would be substantially less than this, because in an actual program setting, the devices would be used, not simply stored. When this usage was taken into account in our Monte Carlo estimation, we found predicted wastage rates to be substantially lower than 10%

At 30 days, wastage levels ranged from 16%–34% in the simulations; actual wastage would be less because field workers would use some of the devices.

### Limitations and Strengths

There are a number of limitations to this study. The first is that we characterized only the distribution of time to discard the packages, based on cumulative exposure to heat of the TTIs. While this is a proxy for heat degradation of oxytocin that would, under programmatic implementation, be stored within these packages, an endpoint of greater interest is the API remaining at various time points over this time range. It is unlikely that the actual API levels of oxytocin stored under similar conditions would follow both the shape and scale parameters of curves for the device packages shown in Figure 3. This is because our empiric estimates of the distribution of time to discard indicates that 100% “failure” occurs on the order of 50 to 60 days, while assays of oxytocin API have generally demonstrated much slower attrition of API under similar temperatures.^4^ Furthermore, the quantitative relationship between deviations from 90% to 110% API, the manufacturer's range of acceptable API, and actual clinical effectiveness of oxytocin is not well understood. For example, a Cochrane Review of the effects on postpartum bleeding of prophylactic oxytocin administered during the third stage of labor included clinical trials in which the dosage of oxytocin varied from 3 to 10 IU, and the mode of delivery included intramuscular injection and intravenous administration.^9^ Thus, it is possible that many of the devices that would need to be discarded as “non-usable” when following the TTI in current use might contain oxytocin that is still within manufacturer specification. Further research is needed to better quantify the optimal level of oxytocin required to produce a clinically sufficient response.

Another limitation is that we did not have sufficient overlap in ambient temperature exposure between phase 1 and phase 2. That is, the mean kinetic temperature to which packages in phases 1 and 2 were exposed rarely overlapped, and thus phase and ambient temperature were too highly correlated for us to determine the impact of the higher mean kinetic temperature exposure during shipment of the phase 2 containers.

This simulation study, however, did use LogTags to document daily variation in temperature exposures from manufacturer to storage in the field under realistic conditions in Ghana and to show how sensitive the time to discard point was to relatively small absolute changes in ambient temperature. Furthermore, our study included a large number of units (N = 550 to 575) examined during 2 different seasons, allowing us to accurately characterize the distributional parameters of the time to discard. By distributing these devices across a set of 23 field workers and collecting characteristics of the storage location, we were also able to further understand the degree to which storage conditions (such as common building construction techniques) influence this distribution of time to discard.

## CONCLUSION

With careful handling of the drug during transport from manufacture to the main storage facility, and careful planning around the appropriate levels of provision to field workers, given known information about rate of use (i.e., the number of units used per day or month), program managers could effectively resupply field workers without refrigeration access with an appropriate level (based on a known usage rate) of Oxytocin-in-Uniject devices (or ampoules of oxytocin) on a monthly basis. Such a schedule would result in high levels of coverage with low overall overage or wastage percentages and would fit with common current resupply schedules for other materials and meeting/supervision schedules.

The TTI used in this study is very conservative with time to discard temperature indicators set at 30°C to 35°C; even lower levels of wastage or more flexible scheduling and resupply schemes could be implemented with a TTI designed to reflect oxytocin stability more closely. Manufacturers could play a role in accelerating programmatic implementation in low-resource settings by developing such a TTI with greater specificity for oxytocin, by improving flexibility in packaging such that TTIs could be affixed to either OIU packages or oxytocin vials (which are more widely available and cost less), and by further elucidating the relationship between temperature exposure and API under various conditions. Oxytocin remains the drug of choice for prevention of postpartum hemorrhage, and OIU has been demonstrated to reduce postpartum hemorrhage by half in home deliveries in Ghana.^8^ The data from this simulation study provide some guidance to program managers as to how this effective intervention might be rolled out at scale and how oxytocin could be used at peripheral health facilities without cold-chain capacity.

## Notes

**Peer Reviewed**

**First published online:** 2014 Jul 10
